# Activation of P2X7 Promotes Cerebral Edema and Neurological Injury after Traumatic Brain Injury in Mice

**DOI:** 10.1371/journal.pone.0041229

**Published:** 2012-07-17

**Authors:** Donald E. Kimbler, Jessica Shields, Nathan Yanasak, John R. Vender, Krishnan M. Dhandapani

**Affiliations:** 1 Department of Neurosurgery, Georgia Health Sciences University, Augusta, Georgia, United States of America; 2 Department of Radiology, Georgia Health Sciences University, Augusta, Georgia, United States of America; Julius-Maximilians-Universität Würzburg, Germany

## Abstract

Traumatic brain injury (TBI) is a leading cause of death and disability worldwide. Cerebral edema, the abnormal accumulation of fluid within the brain parenchyma, contributes to elevated intracranial pressure (ICP) and is a common life-threatening neurological complication following TBI. Unfortunately, neurosurgical approaches to alleviate increased ICP remain controversial and medical therapies are lacking due in part to the absence of viable drug targets. In the present study, genetic inhibition (P2X7−/− mice) of the purinergic P2x7 receptor attenuated the expression of the pro-inflammatory cytokine, interleukin-1β (IL-1β) and reduced cerebral edema following controlled cortical impact, as compared to wild-type mice. Similarly, brilliant blue G (BBG), a clinically non-toxic P2X7 inhibitor, inhibited IL-1β expression, limited edemic development, and improved neurobehavioral outcomes after TBI. The beneficial effects of BBG followed either prophylactic administration via the drinking water for one week prior to injury or via an intravenous bolus administration up to four hours after TBI, suggesting a clinically-implementable therapeutic window. Notably, P2X7 localized within astrocytic end feet and administration of BBG decreased the expression of glial fibrillary acidic protein (GFAP), a reactive astrocyte marker, and attenuated the expression of aquaporin-4 (AQP4), an astrocytic water channel that promotes cellular edema. Together, these data implicate P2X7 as a novel therapeutic target to prevent secondary neurological injury after TBI, a finding that warrants further investigation.

## Introduction

Traumatic brain injury (TBI), a leading cause of mortality and morbidity worldwide, affects over 1.7 million Americans annually [1]. In contrast to primary injuries that occur at the time of impact, secondary pathological processes develop while under supervised medical care and profoundly influence patient outcomes [2]. Cerebral edema, the abnormal accumulation of fluid within the brain, is a life-threatening neurological complication that promotes elevated intracranial pressure (ICP) and leads to clinical deterioration in the hours and days after the initial traumatic event [3], [4]. Increased ICP subsequently promotes brain herniation, limits cerebral blood flow, reduces brain oxygenation, and contributes to poor clinical outcomes [5], [6], [7], [8]; however, the efficacy of neurosurgical approaches to alleviate increased ICP and improve patient prognoses remain limited [9]. Furthermore, effective medical therapies to control ICP are lacking, in part, due to the poorly defined mechanisms that underlie edemic development after TBI.

The innate immune system provides immediate, non-specific defense following infection or tissue injury, although controversy remains as to whether theses response are protective or detrimental after injury. Glia constitutively express receptors involved in cerebral innate immune responses and upon activation, may secrete pro-inflammatory mediators to recruit peripheral immune cells to the site of injury [10]; however, the functional significance and cellular mediators of cerebral innate immune activation remains unresolved. Cellular necrosis correlates with the development of peri-contusional brain edema after TBI and surgical excision of necrotic tissue reduces ICP, decreases patient mortality, and improves neurological outcomes in neurotrauma patients [11], [12], [13]. Thus, necrotic cell death may initiate post-traumatic immune responses. Damage-associated molecular pattern molecules (DAMPs) are multi-functional host proteins that trigger innate immune activation after necrotic injuries. Adenosine 5′-triphosphate (ATP), an important intracellular energy source, is rapidly released into the extracellular space following traumatic or ischemic injuries to function as a non-proteinaceous DAMP [14], [15], [16], [17]. Notably, the accumulation of ATP metabolites within the cerebrospinal fluid (CSF) directly correlated with edemic development and elevated ICP in a neurotrauma patient [18], implicating ATP as an initiator of secondary brain injury after TBI.

Purinergic P2X7 receptors mediate, at least in part, the biological actions of extracellular ATP [19]. Sustained activation of P2X7 with high concentrations of ATP induced the release of biologically-active interleukin-1β (IL-1β) [20], [21], a potent pro-inflammatory cytokine. Notably, IL-1β exhibited a prolonged induction in multiple pre-clinical models of TBI [22], [23], [24], [25], [26], [27] and increased CSF and brain content of IL-1β positively correlated with elevated ICP and unfavorable outcomes in TBI patients [28], [29], [30]. Furthermore, we and others demonstrated that genetic or pharmacological inhibition of IL-1β attenuated both cerebral edema and secondary injury after TBI [26], [31], [32], [33], [34], indicative of a deleterious role for IL-1β after head trauma. However, a mechanistic understanding of post-traumatic IL-1β production remains undetermined and once elucidated, may provide novel opportunities for therapeutic development. As a robust inflammatory response clinically correlates with secondary neurovascular injury after TBI, we hypothesized that activation of P2X7 mediates neurological demise following TBI. Our results implicate P2X7 as a novel therapeutic target to prevent secondary injury after TBI, a finding that warrants further investigation.

## Results

### BBG reduces post-traumatic cerebral edema with an extended therapeutic window

**Figure 1:**
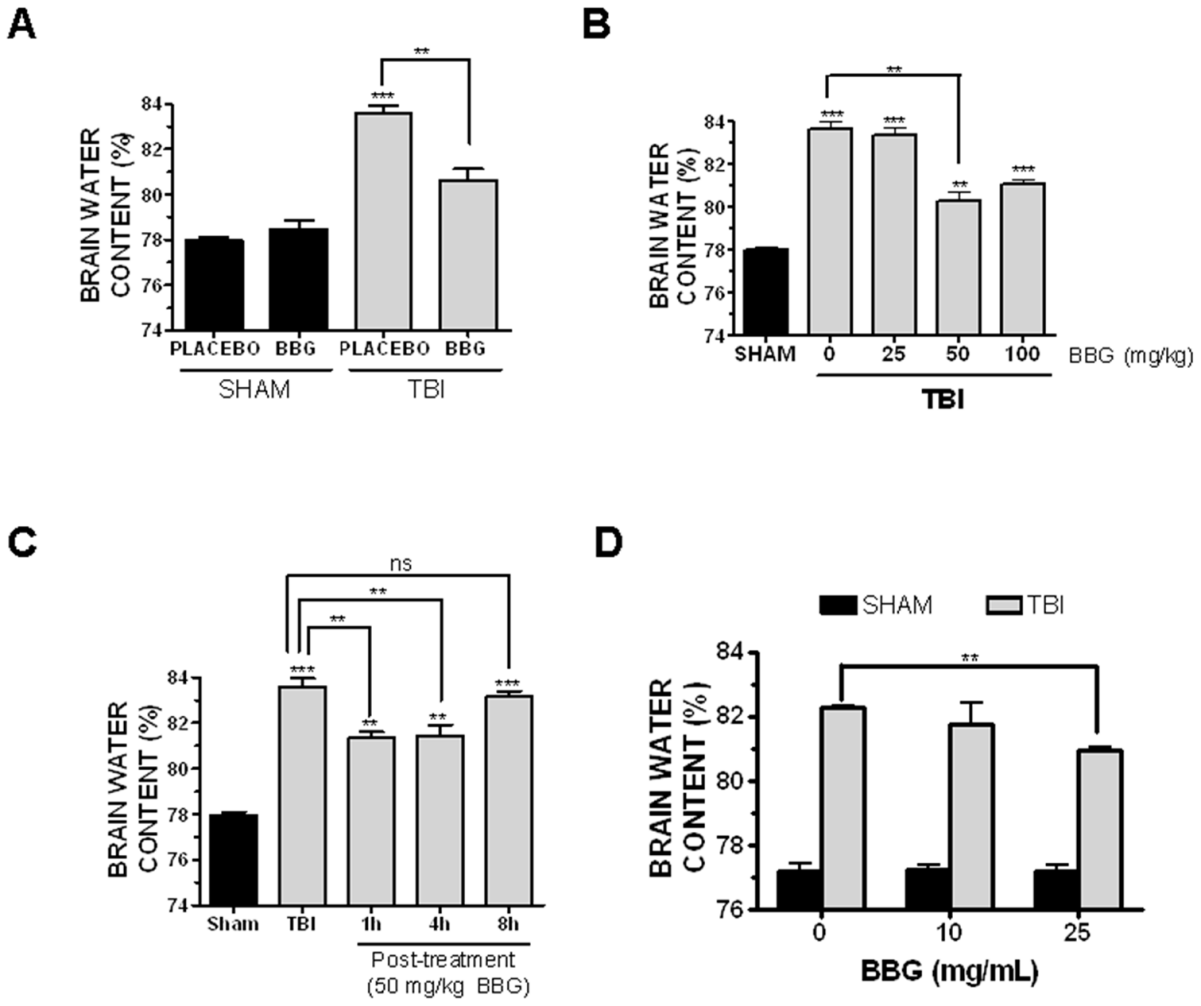
Antagonism of P2X7 reduces cerebral edema after TBI. (**A**) A single intravenous bolus of 50 mg/kg BBG provided 15 minutes prior to TBI, significantly reduced the development of cerebral edema at 24h post-TBI, as measured by brain water content. (**B**) A single intravenous bolus of 50–100 mg/kg BBG administered 0.5h after TBI significantly reduced cerebral edema at 24h post-TBI. (**C**) Administration of a single intravenous bolus of 50 mg/kg BBG reduced cerebral edema when administered 1h or 4h after injury. This effect was lost if post-treatment was delayed beyond 8h from the time of injury. (**D**) Prophylactic treatment with BBG in the drinking water for 7 days reduced edema at 24h post-TBI at a concentration of 25 mg/ml but not 10mg/ml. Comparisons within each hemisphere between different treatments groups were done using a one-way ANOVA followed by Dunnett's post-hoc test (*p<0.05, **p<0.01, ***p<0.001 vs. the ipsilateral hemisphere in sham-operated mice). No significant differences in cerebral edema were observed between groups in the contralateral hemisphere. Data are represented as the mean ± SEM from 5–6 mice/group.

Brain water content, a sensitive measure of cerebral edema, was significantly increased within the ipsilateral cortex at 24h post-TBI (83.6±0.4% brain water content after TBI vs. 77.9±0.2% in sham, p<0.001 vs. sham). A single, intravenous injection of 50 mg/kg BBG at 15 minutes prior to injury attenuated brain water content after TBI (80.6±0.5%; p<0.01 vs. TBI) whereas administration of 25 mg/kg BBG did not significantly reduce edema (83.3%±0.3%; not significantly different from TBI) (**Figure 1a**). Notably, the ability of 100 mg/kg BBG to reduce edema was not significantly different from administration of 50 mg/kg (81.0±0.2%; p<0.001 vs. TBI), suggesting 50 mg/kg was the lowest efficacious dose to limit edemic development after TBI (**Figure 1b**). For all studies, brain water content within the contralateral (uninjured) cortices did not significantly differ between any of the treatment groups (data not shown). Furthermore, administration of BBG alone (50 mg/kg, i.v., 15 minute pre-treatment) did not significantly affect brain water content, as compared to placebo-treated, sham-operated mice (**Figure 1a**), consistent with an injury-specific reduction in edema.

The therapeutic window whereby BBG reduced edemic development was next established. A 1h post-treatment with 50 mg/kg significantly reduced cerebral edema (81.3±0.2%, p<0.05 vs. TBI) to a similar extent as pre-treatment (**Figure 1c**; see **Figure 1a** for comparison). Similarly, a 4h post-treatment effectively attenuated post-traumatic edema (81.4±0.4%, p<0.05 vs. TBI, not significantly different from 1h post-treatment). In contrast, 8h post-treatment with 50 mg/kg was ineffective at reducing edema, as compared to TBI (83.2%±0.2%), suggesting a 4h post-injury therapeutic window.

We next determined whether prophylactic administration of BBG reduces edema. Oral administration of 25 mg/mL BBG via the drinking water for one week prior to injury effectively decreased brain edema after TBI (80.9±0.2%, p<0.01 vs. TBI) (**Figure 1d**). In contrast, 10 mg/mL BBG via the drinking water did not significantly reduce edema, as compared to mice receiving water containing only placebo. As a whole, either prophylactic oral administration or post-injury intravenous administration of BBG effective attenuates brain edema after TBI.

### P2X7−/− mice exhibit reduced cerebral edema after TBI

**Figure 2:**
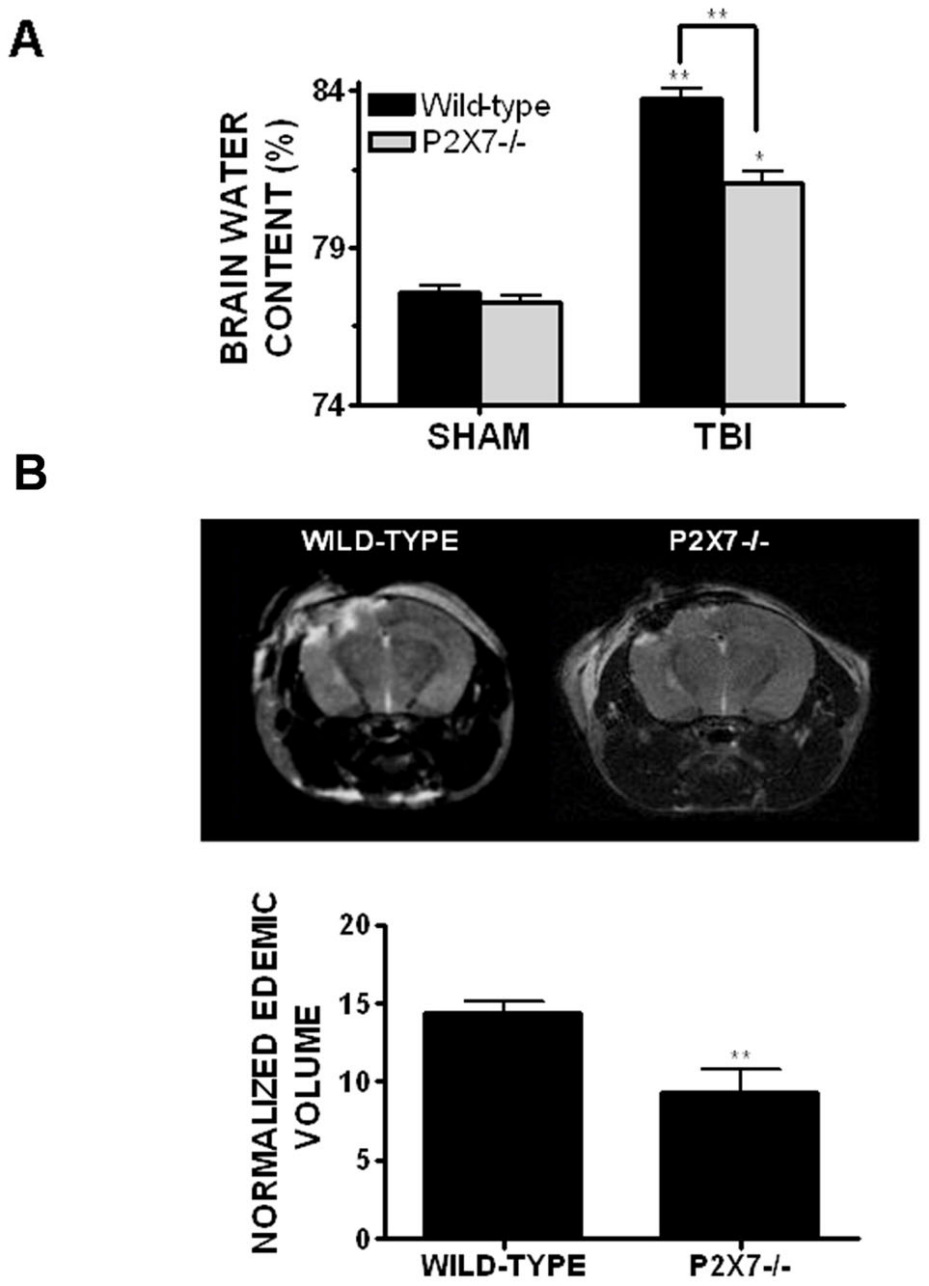
Genetic inhibition of P2X7 attenuates cerebral edema after TBI. (**A**) P2X7−/− mice exhibited a significant reduction in brain water content, as compared to wild-type mice, when assessed at 24h post-TBI. Comparisons within each hemisphere between different treatments groups were done using a one-way ANOVA followed by Dunnett's post-hoc test (* p<0.05 vs. the ipsilateral hemisphere in sham-operated mice). No significant differences in cerebral edema were observed between groups in the contralateral hemisphere. (**B**) P2X7−/− mice displayed attenuated cerebral edema, as compared to wild-type mice, when assessed by MRI. The top panels depict a representative wild-type and a P2X7−/− mouse imaged at 24h post-TBI. Bottom panels represent the mean edemic volume of mice imaged by MRI. Data are represented as the mean ± SEM from six mice/group and were analyzed using a t-test (p<0.01 vs. wild-type).

**Figure 3:**
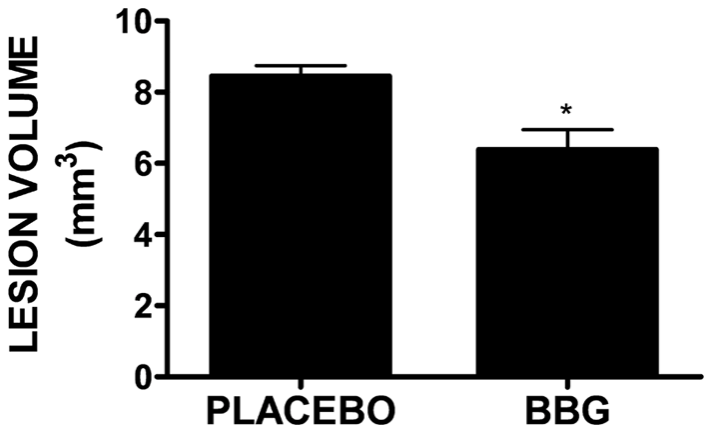
Effect of P2X7 inhibition on cortical lesion volume after TBI. Quantification of cortical lesion volume following placebo or BBG (50 mg/kg, i.p) treatment. Lesion volume is expressed as mm^3^. Data were analyzed using a t-test (*p<0.05 vs. placebo).

BBG is a highly selective inhibitor of P2X7; however, pharmacological agents often exhibit “off-target” or non-specific effects. To validate P2X7 as a potential therapeutic target to reduce brain edema, P2X7−/− mice were utilized. Consistent with data collected after BBG administration, P2X7−/− mice significantly reduced brain water content, as compared to wild-type mice, following TBI (81.0±0.4% in P2X7−/− vs. 83.7±0.3% in wild-type; p<0.01). These findings were supported by the measurement of edemic volume using MRI. P2X7−/− mice exhibited a 36% reduction in edemic volume after TBI, as compared to wild-type mice (14.4±0.7 mm^3^ in wild-type mice vs. 9.2±1.5 mm^3^ in P2X7−/− mice; p<0.01 vs. wild-type) (**Figure 2b**). Brain water content was not significantly different either in sham-operated mice (**Figure 2a**) or in the contralateral hemisphere of wild-type or P2X7−/− mice (data not shown). In line with the reduction in brain edema, inhibition of P2X7 significantly reduced cortical lesion volume after TBI (**Figure 3**). Specifically, lesion volume was decreased from 8.5±0.3 mm^3^ in placebo-treated mice to 6.4±0.6 mm^3^ (p<0.05).

### Brain expression of P2X7 after TBI

**Figure 4:**
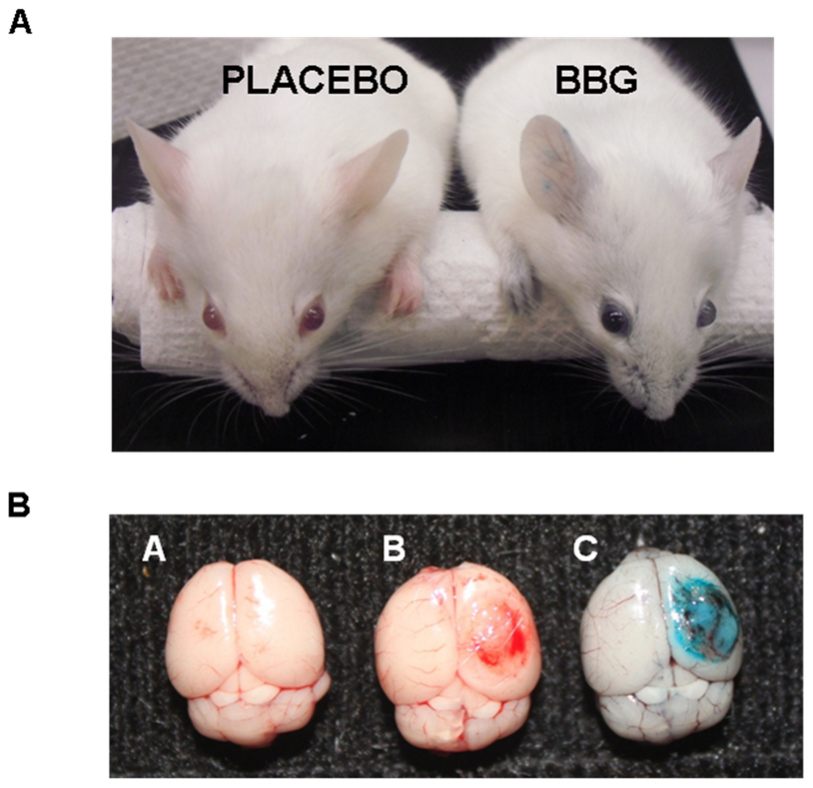
Distribution of BBG after TBI. (**A**) Photograph of representative mice following an intravenous administration of placebo (left) or BBG (50 mg/kg; right). Note the blue appearance in the skin, eyes, ears, paws and tail. (**B**) BBG accumulates in the contused cortex after TBI. Photographs of brains taken from a sham-operated mouse administered placebo (left panel), a mouse administered placebo at 0.5h after TBI (middle panel), or a mouse administered 50 mg/kg BBG via the tail vein at 0.5h post-TBI.

Peripheral administration of BBG reduced brain edema, although the potential tissue and cellular targets of BBG remained unclear. Intravenous administration of 50 mg/kg BBG produced a transient deep blue color over the first 24h within the eyes, nose, ears, and paws (**Figure 4a**), demonstrating wide peripheral distribution throughout the circulatory system. No trace of blue color remained by 72h post-administration. Similarly, oral administration of BBG for one week via the drinking water also produced a faint blue hue in the paws and eyes, albeit to a far lesser extent, as compared to intravenous administration. Consistent with the observed blue appearance, serum levels of BBG reached 383±33.3 μM and 1.73±0.07 mM following intravenous administration of 50 mg/kg and 100 mg/kg, respectively. Whether BBG acted peripherally or centrally after TBI remained unclear. In line with a potential direct effect within the CNS, the brains of mice administered BBG appeared greyish-blue, with blue color observed throughout the cerebral vasculature and brain tissue. Most notably, the contused cortex exhibited a distinct blue color (**Figure 4b**); suggesting BBG can enter the brain and preferentially accumulates at high levels around damaged tissue, presumably following blood-brain barrier disruption.

**Figure 5:**
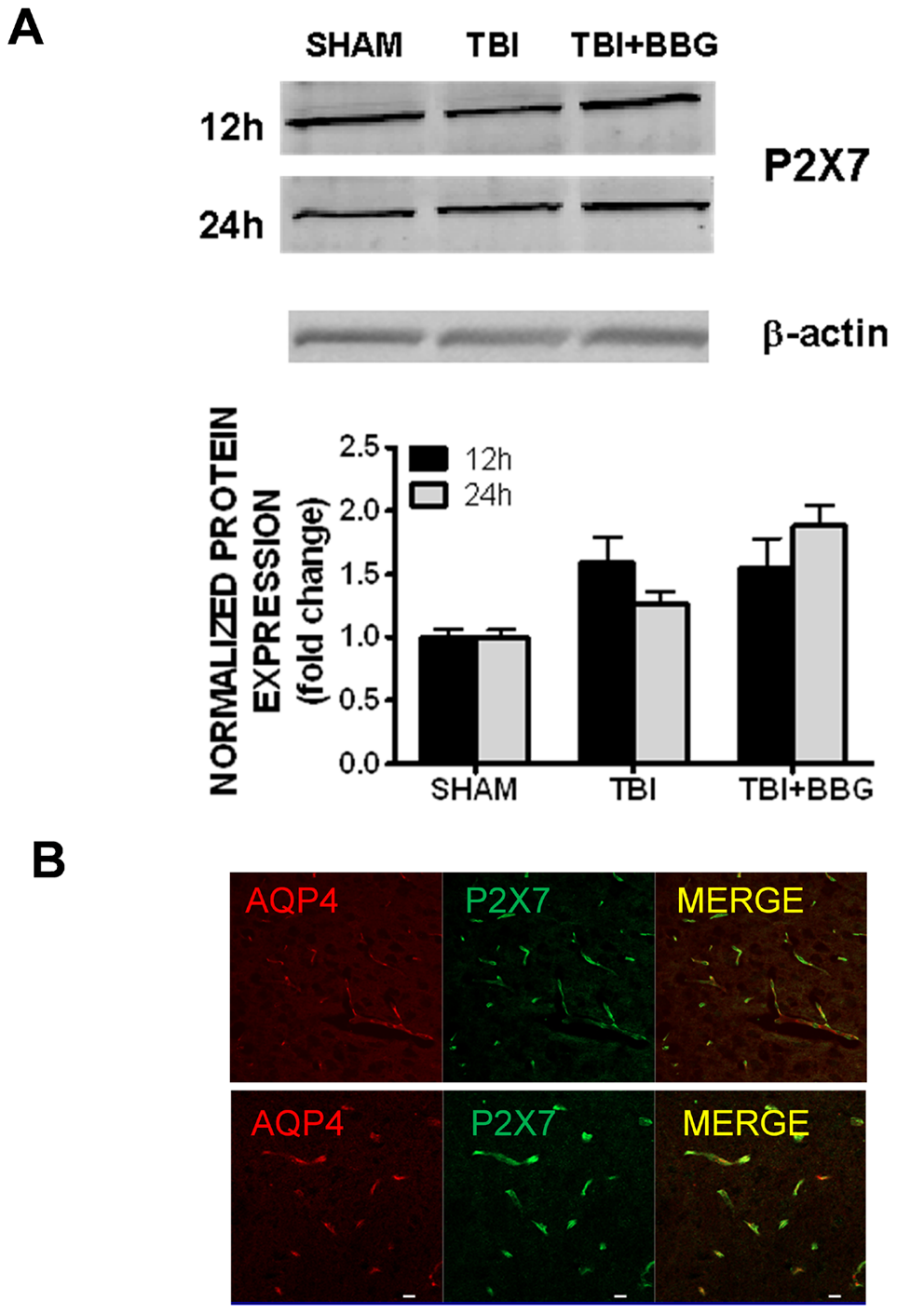
Brain expression of P2X7. (**A**) Representative Western blots (top panel) of P2X7 in the cerebral cortex of mice following sham injury, TBI, or TBI +50 mg/kg BBG. Tissue was collected at 12h or 24h after TBI. Blots were normalized to β-actin to control for equal protein loading between lanes. Data are representative of six mice/group. Densitometric analysis of Western blots (bottom panel) is presented as normalized P2X7 expression. (**B**) Cellular localization of P2X7 in the mouse cerebral cortex by dual immunfluorescence. Brains were immunolabeled for P2X7 (green) and AQP4 (red), a marker of astrocytic endfeet. Confocal images (top panel, 25x objective; bottom panel, 40x objective) were obtained from the pericontusional cortex. Scale bar  = 20 µm.

The cellular expression pattern of P2X7, the presumed cellular target of BBG action, was next assessed within the brain. P2X7 was basally expressed within the cerebral cortex, as demonstrated by Western blotting; however, expression was not increased following TBI, as compared to sham-operated mice (**Figure 5a**). Immunohistochemical analysis revealed that P2X7 strongly co-localized with the astrocytic end foot marker, aquaporin-4 (AQP4) (**Figure 5b**) whereas dual labeling was not observed with either markers of microglia or neurons (data not shown). These data implicate astrocytes as key mediators of the biological actions of P2X7 and as a possible cellular target of BBG after TBI.

### P2X7 mediates IL-1β expression after TBI

**Figure 6:**
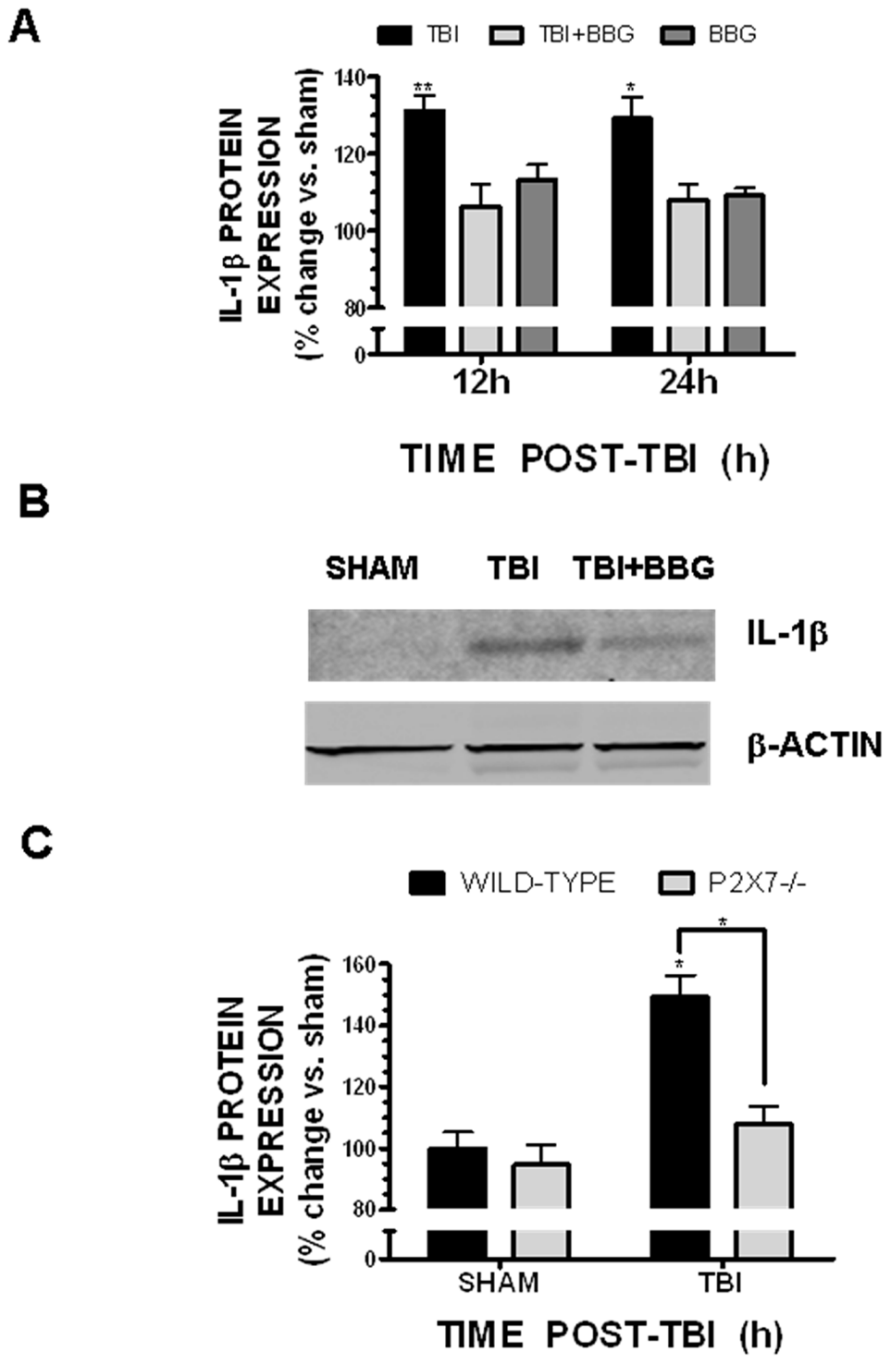
Inhibition of P2X7 attenuates post-traumatic IL-1β expression. A single intravenous bolus of 50–100 mg/kg BBG administered 0.5h after TBI significantly reduced peri-contusional IL-1β expression, as assessed by (**A**) EIA and by (**B**) Western blotting at 12h or 24h post-injury. (**C**) IL-1β was quantified by EIA at 24h post-injury in wild-type or P2X7−/− mice. In panels A and C, data are represented as IL-1β expression as a % of sham expression levels. In panel B, data was normalized to β-actin to control for equal protein loading between lanes. Data are representative of 6–8 mice/group. Data were analyzed with One-Way ANOVA followed by Dunnett's post-hoc test (* p<0.05, ** p<0.01 vs. sham operated mice).

Increased expression of the pro-inflammatory cytokine, IL-1β, clinically correlates with the development of cerebral edema after brain injury. Consistent with the ability of BBG to attenuate post-traumatic edema, post-treatment with 50 mg/kg BBG significantly reduced the expression of biologically mature IL-1βwithin the pericontusional cortex at 12h and 24h after injury, as assessed by either enzyme-linked immunoassay (EIA) (**Figure 6a**) or by Western blotting (**Figure 6b**). TBI increased IL-1β expression by 30.9±1.1% and 28.8±5.5% over sham-operated mice at 12h (p<0.01 vs. sham) and 24h (p<0.05 vs. sham), respectively. Post-treatment with 50 mg/kg BBG reduced the post-traumatic expression of IL-1β to 6.8±6.3% and 8.0±4.0% over sham (p<0.01 and p<0.05 vs. TBI at 12h and 24h, respectively; not significantly different from sham at either timepoint). Notably, administration of 50 mg/kg BBG alone had no significant effect on the basal expression of IL-1β, as compared to sham-operated mice. In line with these observations, a significant reduction in post-traumatic IL-1β expression was observed in the pericontusional cortex of P2X7−/− mice, as compared with wild-type mice (**Figure 6c**). Specifically, IL-1β expression was increased by 49.3±7.1% at 24h post-TBI in wild-type mice (p<0.05 vs. sham). In contrast, a 7.8±5.9% increase was observed in P2X7−/− mice (p<0.05 vs. wild-type TBI; not significantly different from sham). No significant differences in basal IL-1β expression were detected between genotypes, suggesting an injury-specific effect of P2X7 knockout.

### P2X7 mediates glial reactivity after TBI

**Figure 7:**
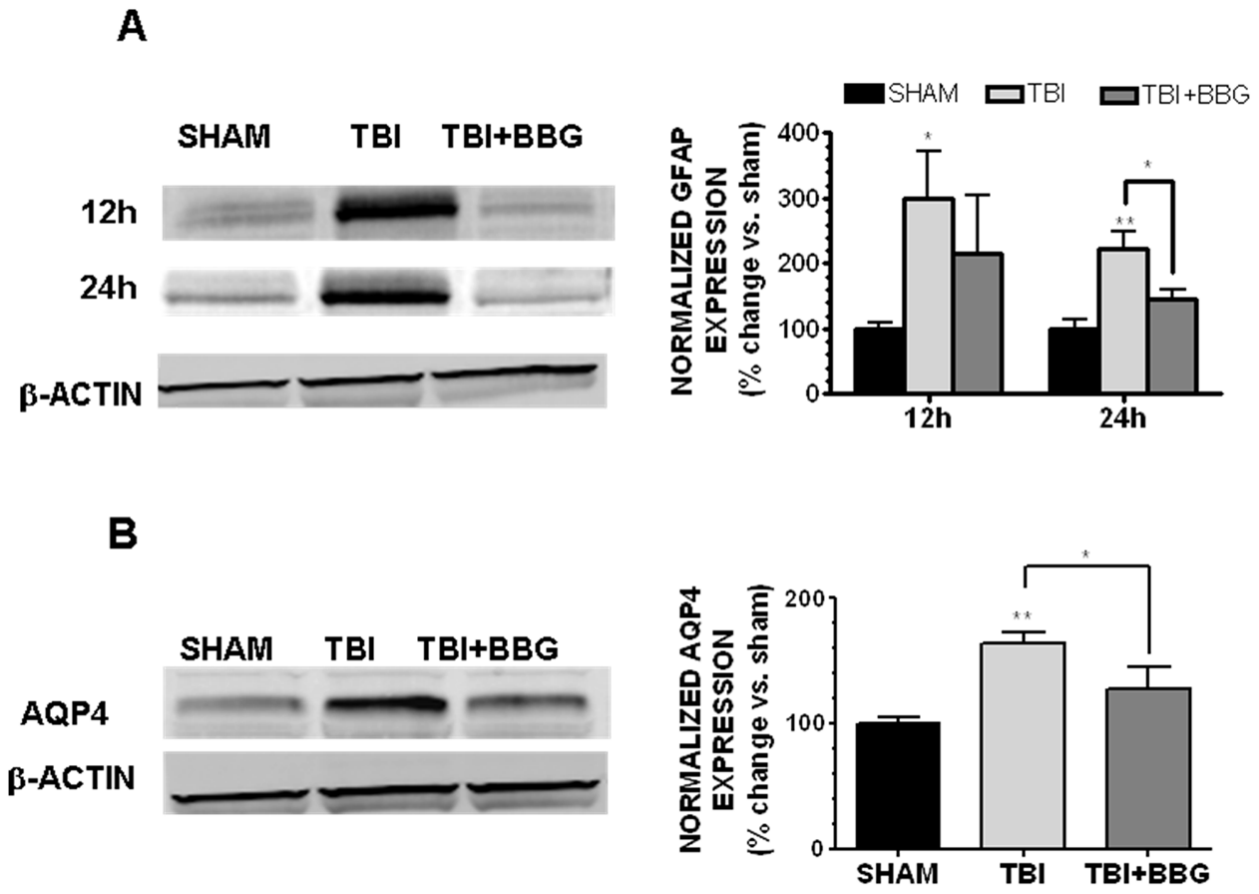
BBG attenuates glial activation. (**A**) Representative Western blot (left panel) of cortical GFAP expression taken at 12h or 24h after sham injury, TBI, or TBI +50 mg/kg BBG. (**B**) Representative Western blot (left panel) of AQP4 in the cerebral cortex of mice at 12h following sham injury, TBI, or TBI +50 mg/kg BBG. Densitometric analysis of Western blots (right panels) is presented as either GFAP or AQP4 expression following normalization to β-actin, which was used to control for equal protein loading. Data (mean ± SEM) are representative of six mice/group from three independent experiments (n = 3/group in each experiment) and are expressed as % change vs. sham. Data were analyzed by One-Way ANOVA followed by Dunnett's post-hoc test (* p<0.05, ** p<0.01 vs. sham operated mice).

IL-1β induces reactive astrogliosis after TBI; therefore, the ability of BBG to attenuate the expression of GFAP, a hallmark of gliosis, was next assessed. GFAP expression was significantly increased by 299.7±72.2% within the peri-contusional cortex (p<0.05 vs. sham) and 222.0±28.6% (p<0.01 vs. sham) of sham-operated mice at 12h and 24h post-TBI, respectively (**Figure 7a**). Post-treatment with 50 mg/kg BBG reduced GFAP expression to 216.1±88.6% (not significantly different from either sham or TBI) and 145.3±15.2% (p<0.05 vs. TBI, not significantly different from sham) of expression levels in sham-operated mice at 12h and 24h, respectively.

Consistent with the inhibitory effect of BBG on post-traumatic cerebral edema and glial reactivity, BBG attenuated the expression of the astrocytic water channel, AQP4, after TBI. AQP4 protein expression was increased within the pericontusional context at 12h (1.7±0.1 fold increase; p<0.01 vs. sham) and at 24h (1.5±0.1 fold increase; p<0.05 vs. sham) after TBI. Intravenous administration of 50 mg/kg BBG at 0.5h post-injury attenuated the TBI-induced increases in AQP4 expression (1.3±0.2 and 1.1±0.1 fold increase vs. sham at 12h and 24h, respectively; p<0.05 vs. TBI, not significant different from sham) (**Figure 7b**).

### BBG improves neurobehavioral outcomes after TBI

**Figure 8:**
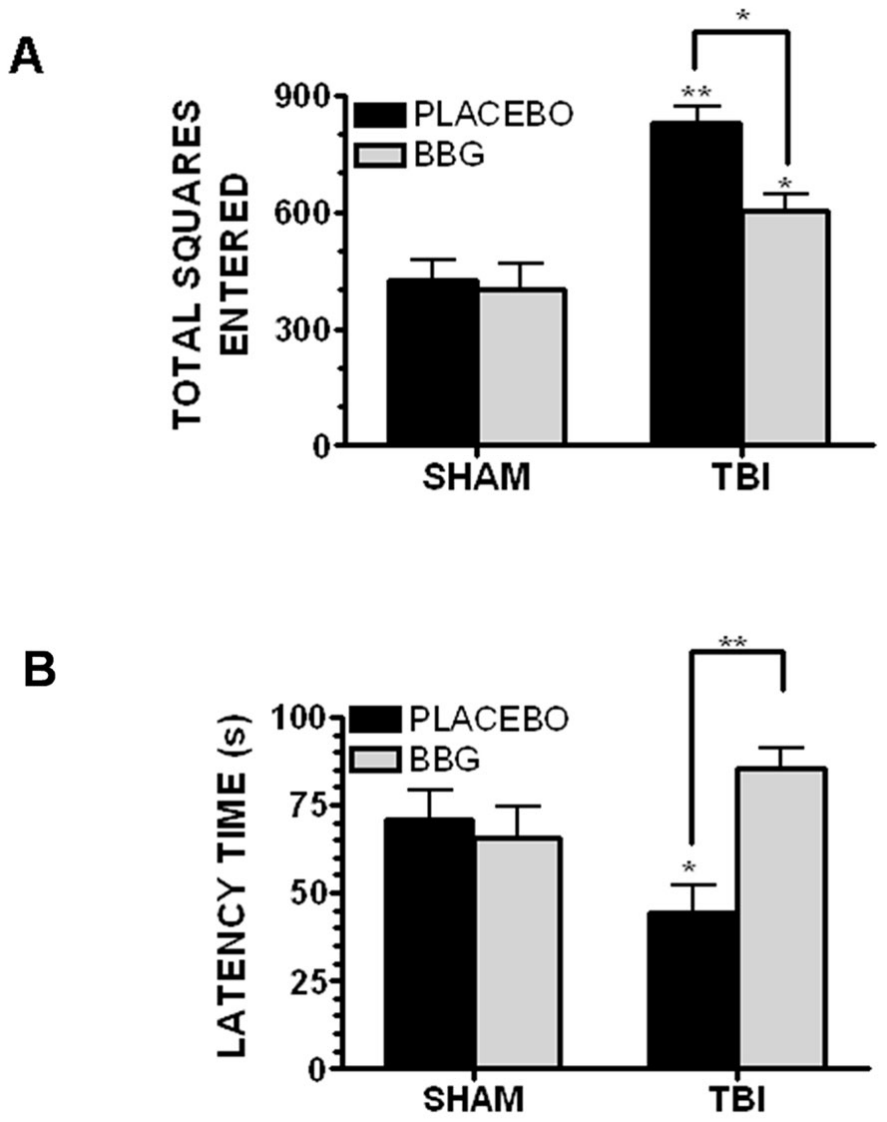
BBG improves neurological outcomes after TBI. Post-injury administration of 50 mg/kg BBG significantly attenuated (**A**) post-traumatic hyperlocomotion following TBI in the open field test and (**B**) time to first immobility in the forced swim test, a sensitive estimate of depressive like behavior, as compared to placebo-treated mice. Data are expressed as the mean ± SEM from 10–12 mice/group and were compared by One-Way ANOVA followed by Dunnett's post-hoc test (* p<0.05, ** p<0.01 vs. sham operated mice).

Depression, anxiety, and cognitive dysfunction are frequent co-morbidities after a TBI. A significant increase in open-field hyperlocomotion (total number of squares entered) was observed following TBI (p<0.01 vs. sham) (**Figure 8a**). Administration of 50 mg/kg BBG partially attenuated post-traumatic hyperlocomotion by ∼50% (p<0.05 vs. sham and TBI). In contrast, BBG administration had no significant effect on basal activity in sham-operated mice. Despite a reduction in overall activity, BBG failed to significantly influence the degree of thigmotaxis, a measure of anxiousness (data not shown). Following TBI, mice exhibited a reduced time to latency to develop behavioral despair, a measure of depression, using the forced swim test (**Figure 8b**). Sham-operated mice displayed a latency of 70.8±8.3s whereas TBI reduced this time to 44.5±7.4s (p<0.05 vs. sham). Post-injury administration of 50 mg/kg BBG significantly increased the latency time to 85.4±5.5s (p<0.01 vs. TBI, not significantly different from sham). Notably, BBG administration did not significantly change the latency time in sham-operated mice, suggesting an injury specific effect.

## Discussion

Preventative measures reduce the incidence and/or severity of TBI, yet one-third of hospitalized TBI patients die from injuries that are secondary to the initial trauma. The development of post-traumatic edema promotes clinical deterioration and worsens long-term outcomes, at least in part, by limiting cerebral perfusion, by increasing brain herniation, and by increasing the manifestation of neurological impairments such as headaches, anxiety, depression, sleep disturbances, cognitive dysfunction and appetite loss [3], [4], [35], [36]. Thus, elucidation of the cellular mechanisms of neurological injury may permit the development of efficacious therapeutics to improve patient outcomes after TBI.

In the present study, genetic (P2X7−/−) or pharmacological (BBG) inhibition of P2X7 reduced secondary brain injury and improved functional outcomes after a moderate TBI in mice. BBG, a FDA-approved, water soluble, structural and functional analogue of FD&C blue dye No. 1 (also called Brilliant blue FCF or E133), is a widely used food additive and coloring agent that exhibits no toxicity at doses up to 1g/kg/d in humans [37]. Herein, BBG reduced peri-contusional IL-1β, limited AQP4 expression, attenuated edemic development, and improved neurobehavioral outcomes. These beneficial effects were observed whether BBG was intravenously administered as a single bolus up to four hours after injury or chronically administered via the drinking water prior to injury. Thus, clinically safe doses of BBG may reduce neurological injury after TBI, either via a clinically-implementable post-injury temporal window or via prophylactic administration.

Cellular edema is the predominant form of edema during the acute and sub-acute phase after TBI [38], [39]. Astrocytic swelling, a characteristic feature of cellular edema, commenced within the first hours after head trauma in humans [38], [39] and glial activation temporally paralleled edemic development in pre-clinical models of TBI [40], [41]. Furthermore, increased serum and CSF levels of the activated astrocyte markers, S100β and GFAP, directly correlated with patient outcomes after TBI [29], [42], [43], supporting a possible role for astrocytes in the genesis of secondary neurovascular injury; however, controversy remains as to whether astrocytes exert beneficial and/or detrimental functions after brain injury [44]. Along these lines, astrocytes are the predominant cell type within the neurovascular unit, providing trophic support for neurons, regulating cerebral blood flow, and maintaining ionic and neurotransmitter homeostasis under physiological conditions. Conversely, astrocytes may generate cerebral innate immune responses after injury or infection, releasing pro-inflammatory mediators [10].

AQP4, a bidirectional water channel expressed in the perivascular end feet of astrocytes, mediated glial swelling *in vitro* and was associated with the development of cellular edema after TBI in humans and rodents [45], [46]. Although causative studies remain unperformed after neurotrauma, attenuated swelling of pericapillary astrocytic foot processes, decreased cellular edema, and reduced mortality were observed in AQP4-deficient mice after ischemic stroke or after acute water intoxication [47]. Additionally, genetic deletion of AQP4 attenuated astrocytic migration and glial scar formation, implicating AQP4 as a potential therapeutic target to restrict deleterious astrocytic responses to injury [48]. Unfortunately, clinically-efficacious drugs to inhibit AQP4 expression/function do not currently exist, at least in part, due to the limited understanding of AQP4 regulation at the cellular level. Notably, we and others recently identified IL-1β as a positive regulator of AQP4 expression in cultured astrocytes and in the mouse cerebral cortex [26], [49]. IL-1β expression is rapidly increased following brain insults and functionally promoted reactive astrogliosis after penetrating brain injury [50]. Furthermore, elevated concentrations of IL-1β in the CSF of TBI patients correlated with an unfavorable clinical outcome [29], [30]. Based on these findings, we hypothesized that strategies which reduce post-traumatic IL-1β may effectively limit neurovascular injury after TBI.

IL-1β is synthesized as a biologically inactive 31-kDa precursor protein that requires proteolytic cleavage to generate the mature, biologically-active 17.5 kDa protein [51]. Expression of caspase-1 (also called interleukin-1 converting enzyme; ICE), the principal enzyme involved in the processing of pro-IL-1β into the mature IL-1β form, was upregulated within the rat forebrain after fluid percussion injury [52]. Activated caspase-1 was strongly increased in brain tissue resected from both pediatric and adult TBI patients whereas pro-caspase-1 exhibited a decrease in expression as compared to control patients [53]. Furthermore, activated caspase-1 was elevated within the CSF of pediatric TBI patients, an observation that directly correlated with a concomitant increase in IL-1β expression and a reduction in pro-IL-1β in these same patients [53]. Functionally, genetic or pharmacological inhibition of caspase-1 reduced secondary tissue damage after experimental TBI in mice [53]. Taken together, these findings suggest clinical significance for caspase-1 activation after TBI and imply therapeutic targeting of caspase-1 pathway may improve outcomes.

The precise cellular mechanisms underlying caspase-1 activation remain poorly defined; however, repetitive or prolonged exposure to high concentrations of ATP increased the activation and the externalization of caspase-1 and promoted the formation of a large membrane pore required for the extracellular release of IL-1β [54], [55]. ATP, an intracellular energy source under physiological conditions, is rapidly released into the extracellular space after traumatic or ischemic injuries [14], [15], [16], [17]. Although the functional significance remains poorly defined, the release of extracellular ATP promoted secondary tissue damage after traumatic spinal cord injury [17]. Furthermore, elevated levels of ATP metabolites within the CSF of a head trauma patient correlated with edemic development and elevated ICP [18], implying a detrimental role for purinergic signaling after neurological injury.

The biological actions of ATP are mediated, at least in part, by activation of either metabotropic P2Y receptors or ionotropic P2X receptors [14]. Among the purine receptor family members, P2X7 is a low-affinity receptor that preferentially responds to sustained elevations in ATP such as those which occurs after trauma, suggesting P2X7 possesses the optimal biophysical properties for mediating the detrimental actions of ATP after a brain injury. Herein, P2X7 specifically co-localized within astrocytic end feet within the brain, directly overlapping with the expression of AQP4. Consistent with a report showing extracellular ATP induced stellation and increased GFAP expression in astrocyte cultures [56], clinically-achievable doses of BBG decreased IL-1β production, reduced astrocytic activation, as assessed by GFAP expression, attenuated AQP4 expression, and limited cerebral edema after TBI in mice. Given the importance of cerebral edema and elevated ICP in patient mortality and long-term morbidity after TBI, P2X7 antagonism may improve acute clinical outcomes following TBI.

Increased rates of depression, aggression, anxiety, and cognitive dysfunction are observed over the first year in over 51% of TBI survivors [57]. Interestingly, patients with idiopathic intracranial hypertension, a neurological disorder characterized by non-traumatic elevations in ICP, exhibited higher rates of developing depression and anxiety, as compared to matched control patients [58]. These clinical findings suggested post-traumatic elevations in ICP could directly induce psychiatric co-morbidities. Unfortunately, a recent meta-analysis of 223 pre-clinical trials failed to identify any single intervention that significantly improved these neurological outcomes after TBI [59]. IL-1β, which clinically correlates with elevated ICP after TBI [28], [30], [60], is implicated in the pathophysiology of depression and anxiety [61], [62], [63], [64] and in neuronal cell death and cognitive dysfunction after experimental TBI [31], [32], [33], [34], [65]. Thus, IL-1β may provide a key mechanistic bridge between acute traumatic injury and long-term neurological outcomes. Consistent with this notion, post-injury administration of clinically-relevant doses of BBG that reduced IL-1β expression and limited post-traumatic edema, attenuated the manifestation of depressive-like and improved performance in the open-field task, a measure of cognitive function and/or anxious behavior, after TBI. This finding is in line with a report showing P2X7−/− mice exhibited an anti-depressive-like profile and increased responsiveness to antidepressant drugs under basal conditions, as compared to wild-type mice [66]. The novel findings presented herein provide support for the notion that acute neuroinflammatory mediators contribute to elevations in ICP as well as influence the development of subsequent neurobehavioral outcomes after TBI.

Several caveats of this study warrant further consideration. Although considered a highly selective P2X7 antagonist, BBG also can inhibit both P2X2 and P2X5, albeit less potently than at P2X7 [67]. Despite our data showing P2X7−/− mice exhibit similar responses to BBG-treated mice, we cannot exclude the possibility that off-target effects on receptors other than P2X7 mediated the beneficial actions of BBG. Similarly, it remains unclear whether BBG penetrates the blood-brain barrier. We observed a significant accumulation of BBG within the tissue adjacent to the contusion, suggesting BBG could possibly act at the level of the CNS. Nonetheless, we cannot eliminate the possibility that BBG may also act on peripheral immune cells that express P2X7, produce pro-inflammatory mediators, and infiltrate into brain tissue after TBI. Future work by our group using cell-type specific knockout of P2X7 (e.g. astrocyte-specific P2X7 knockout) will attempt to address this issue in detail.

In conclusion, our data suggests a novel, causative role for the low-affinity ATP receptor, P2X7, in the development of cerebral edema and neurological injury after TBI. These findings also identify BBG, a drug that is well-tolerated in humans, in the treatment of cerebral edema and neurological deterioration following TBI using a clinically-feasible therapeutic window. Given the dearth of medical treatment options to limit elevated ICP and reduce co-morbid psychiatric deficits following head trauma, further exploration of P2X7 may be warranted.

## Materials and Methods

### Controlled Cortical Impact

The Committee on Animal Use for Research and Education at Georgia Health Sciences University approved all animal studies (Protocol Approval #2010–0168), in compliance with NIH guidelines. Adult male CD-1 (Charles River, Wilmington, MA), C57Bl/6, or P2X7 knockout (P2X7−/−; Jackson Laboratories) mice were anesthetized with xylazine (8 mg/kg)/ketamine (60 mg/kg) and subjected to a sham injury or controlled cortical impact, per our laboratory [26], [68]. Briefly, mice were placed in a stereotaxic frame (Amscien Instruments, Richmond, VA, USA) and a 3.5 mm craniotomy was made in the right parietal bone midway between bregma and lambda with the medial edge 1 mm lateral to the midline, leaving the dura intact. Mice were impacted at 4.5 m/s with a 20 ms dwell time and 1 mm depression using a 3 mm diameter convex tip, mimicking a moderate TBI. Sham-operated mice underwent the identical surgical procedures, but were not impacted. The incision was closed with VetBond and mice were allowed to recover. Body temperature was maintained at 37°C using a small animal temperature controller throughout all procedures (Kopf Instruments, Tujunga, CA, USA).

### Treatments

For acute drug administration studies, placebo (phosphate-buffered saline, PBS) or 25–100 mg/kg brilliant blue G (BBG; 100% pure, Acros Organics), a highly specific and clinically-useful P2X7 antagonist [67], was administered via the tail vein 15 minutes prior to or up to 8 hours after TBI. For prophylactic studies, mice were group housed in standard cages with mouse chow provided *ad libitum.* Placebo treated cages received 2% sucrose (w/v in tap water) whereas BBG treated cages received 25 mg/mL BBG in 2% sucrose water. Oral continued throughout the duration of the study. Both intravenous and oral drug administration were well-tolerated and differences in locomotor activity or body weights were not observed, as compared to non-experimental mice fed a standard diet of chow and tap water.

### Assessment of cerebral edema

Brain water content (BWC), a sensitive measure of cerebral edema, was quantified using the wet-dry method, as detailed by our group [26], [69]. At 24h post-injury, a time-point associated with significant edema formation after experimental TBI [26], [70], [71], BWC was estimated in 3 mm coronal sections of the ipsilateral cortex (or corresponding contralateral cortex), centered upon the impact site. Tissue was immediately weighed (wet weight), then dehydrated at 65°C. The sample was reweighed 48h later to obtain a dry weight. The percentage of tissue water content was calculated using the following formula: BWC  =  [(wet weight)-(dry weight)/wet weight] * 100.

### Determination of lesion size

Cortical lesion area was quantified by an investigator blinded to experimental conditions, as described by our laboratory [26]. Briefly, serial coronal sections were digitized using a Zeiss Axiophot microscope using a 2.5X objective and imported into the OsiriX v2.7.5 32-bit program. A region of interest was drawn along the perimeter of the injured cortex and lesion volume was calculated and expressed as mm^3^.

### Magnetic resonance imaging (MRI)

Non-invasive determination of brain edema was performed using a horizontal 7.0T BioSpec MRI spectrometer (Bruker Instruments) equipped with a 8.9 cm micro-imaging gradient insert (100 gauss/cm). For all studies, anesthetized mice were positioned with the MR scanner. Breathing was controlled at 35 respirations/minutes and core body temperature was maintained at 37°C using a recirculating water bath. High-resolution T2-weighted (T2W) images and diffusion-weighted images (DWI) were acquired during each session, using a surface coil developed in-house. Two T2W image volumes were acquired using a 2D RARE sequence (TR/TEeffective = 2800/56ms; RARE factor = 8; FOV = 25.6×25.6mm; Matrix = 256×256; slice thickness = 0.5 mm; slice gap = 0.5mm; 20 slices; 5 NEX), offset by 0.5 mm to allow for collection of data without a gap. A diffusion tensor imaging (DTI) sequence was used for calculation of an apparent diffusion coefficient (ADC) map (TR/TE = 2800/60ms; FOV = 25.6×25.6mm; Matrix = 128×128; slice thickness = 0.8mm; slice gap = 0.2mm; 3 b = 0 images; six directions; Delta = 14 ms; delta = 7 ms; b value = 1200 sec mm2; 12 slices; 16 repetitions). Apparent diffusion coefficient (ADC) values, which are rotationally invariant, reduces systemic bias that may be introduced by variation in animal orientation. After normalizing image intensities and ADC values between pre- and post-TBI images, T2W images were segmented according to brightness to identify regions of edema, and the edemic volume was computed. A Region of Interest (ROI) was superimposed on the ADC map to characterize the edema as cellular or vasogenic.

### Assessment of neurological injury

Mice were housed on a 12-hour light-dark cycle in a climate-controlled vivarium throughout the experiment. At 72h after injury, a 2h undisturbed habituation period was implemented prior to the initiation of behavioral testing. All tests were digitally recorded using a video camera fixed above the floor and scored by an investigator who was blinded to experimental conditions. For the open field test, animals were placed into a 14×14 inch box with 2×2 inch squares, as detailed by our laboratory [26], [69], [72]. Locomotion, as assessed by the number of squares entered over the 5-minute testing period, was defined as the nose and two forepaws entering a new square. Directly following the open field test, mice were placed in a cylinder filled with 13 cm of 37°C water. Behavioral despair, defined as the time when an animal ceases to attempt to escape from a stressful situation, was quantified as the latency time to the first total immobility in the forced swim. This event was defined as all 4 paws and head remaining motionless in the water.

### Western blotting

Whole cell lysates were prepared from 3 mm coronal sections centered upon the site of impact. A 1-mm micropunch was collected from the pericontusional cortex or from the corresponding contralateral hemisphere. Tissue was placed in complete RIPA buffer, sonicated, and centrifuged for 10 minutes at 14,000×*g* at 4°C. Protein concentrations were quantified using a BCA protein assay kit (Pierce, Rockford, IL). 30 μg of protein were resolved on a 4–20% sodium dodecyl sulfate-polyacrylamide gel and transferred onto a polyvinylidene difluoride (PVDF) membrane. Blots were incubated overnight at 4°C in primary antibody [(1∶200 anti-P2X7, Alomone Labs, Jerusalem, Israel), (1∶400 anti-AQP4 antibody, Santa Cruz Biotechnology, Santa Cruz, CA), (1∶200 anti-GFAP antibody, Dako, Carpinteria, CA), (1∶5000 anti-IL-1β antibody, National Cancer Institute, clone 3ZD, lot 1), or (1∶2000 anti-β actin, Abcam, Cambridge, MA) followed by a 2h incubation with an Alexa Fluor-tagged secondary antibody at room temperature, per our laboratory [73], [74], [75], [76], [77]. Blots were visualized using a Li-Cor Odyssey near-infrared imaging system and densitometry analysis was performed using Quantity One software (Bio-Rad, Foster City, CA).

### Immunohistochemistry

Deeply anesthetized mice were perfused with saline, followed by fixation with 4% paraformaldehyde in 0.1 M phosphate buffer (pH 7.4). Brains were post-fixed overnight in paraformaldehyde followed by cryoprotection with 30% sucrose (pH 7.4) until brains permeated. Serial coronal sections (12 μM) were prepared using a cryostat microtome (Leica, Wetzlar, Germany) and directly mounted onto glass slides. Sections were incubated at room temperature with 10% normal donkey serum in phosphate-buffered saline containing 0.4% Triton X-100 for 1 h, followed by incubation with the primary antibody [P2X7 (1∶100), AQP4 (1∶200), or GFAP (1∶500)] overnight at 4°C. Sections were then washed and incubated with the appropriate Alexa Fluor-tagged secondary antibody. Omission of primary antibody served as a negative control.

### Confocal microscopy

Immunofluorescence was determined using a LSM510 Meta confocal laser microscope (Carl Zeiss), as described by our group [26], [68]. Cellular co-localization was determined in Z-stack mode using 63X oil immersion Neofluor objective (NA 1.3) with the image size set at 512×512 pixels. The following excitation lasers/emission filters settings were used for various chromophores: argon2 laser was used for Alexa Fluor 488, with excitation maxima at 490 nm and emission in the range 505–530 nm. A HeNe1 laser was used for Alexa Fluor 594 with excitation maxima at 543 nm and emission in the range 568–615 nm. Z-stacks (20 optical slices) were collected at optimal pinhole diameter at 12-bit pixel depth and converted into three-dimensional projection images using LSM510 Meta imaging software.

### Statistical analysis

The effects of treatments were analyzed using a one-way analysis of variance (ANOVA) followed by Dunnett's post hoc test. Results are expressed as mean ± SEM. A p<0.05 was considered to be statistically significant.
